# Innate Immune Cells and Their Contribution to T-Cell-Based Immunotherapy

**DOI:** 10.3390/ijms21124441

**Published:** 2020-06-22

**Authors:** Pierpaolo Ginefra, Girieca Lorusso, Nicola Vannini

**Affiliations:** 1 Laboratory of Immunosenescence and Stem Cell Metabolism, Department of Oncology, Ludwig Cancer Institute, University of Lausanne, 1066 Epalinges, Switzerland; pierpaolo.ginefra@unil.ch; 2 Experimental and Translational Oncology, Department of Oncology, Microbiology, Immunology (OMI), Faculty of Science and Medicine, University of Fribourg, 1700 Fribourg, Switzerland; girieca.lorusso@unfr.ch

**Keywords:** immunotolerance, immunotherapy, innate immunity, adaptive immunity, myeloid cells

## Abstract

In recent years, immunotherapy has become the most promising therapy for a variety of cancer types. The development of immune checkpoint blockade (ICB) therapies, the adoptive transfer of tumor-specific T cells (adoptive cell therapy (ACT)) or the generation of T cells engineered with chimeric antigen receptors (CAR) have been successfully applied to elicit durable immunological responses in cancer patients. However, not all the patients respond to these therapies, leaving a consistent gap of therapeutic improvement that still needs to be filled. The innate immune components of the tumor microenvironment play a pivotal role in the activation and modulation of the adaptive immune response against the tumor. Indeed, several efforts are made to develop strategies aimed to harness innate immune cells in the context of cancer immunotherapy. In this review, we describe the contribution of innate immune cells in T-cell-based cancer immunotherapy and the therapeutic approaches implemented to broaden the efficacy of these therapies in cancer patients.

## 1. Introduction

Over the last decade, cancer immunotherapy, such as immune checkpoint blockade (ICB) and adoptive cell therapy (ACT), has emerged as revolutionary advances in cancer treatment. The impact of these new approaches led to the assignment of the Nobel Prize for Medicine to James Allison and Tasuku Honjo in 2018 for their discoveries on negative immune regulation and the development of ICB. Compared to conventional anticancer therapies that directly target the malignant cells, immunotherapy exploits the patient’s own immune cells to recognize and eventually eliminate the tumor [1,2]. ICB elicits activation of endogenous cancer-specific T cells by antibody-mediated blockade of the checkpoint inhibitor molecules CTLA-4, programmed cell death protein-1 (PD-1) and programmed death-ligand-1 (PD-L1). CTLA-4 is expressed by activated T cells and regulatory T cells (Treg) [3] and induces immune tolerance and T cell anergy upon binding to CD80 and CD86 expressed by antigen-presenting cells (APC) [4]. Similarly, PD-1 is expressed by T cells and binds specific ligands called PD-L1 and PD-L2 presented by cancer cells and other immune components of the tumor microenvironment. PD-1 interaction with its ligands leads to T cell receptor (TCR) signaling inactivation and T cell exhaustion [4]. Since both CTLA-4 and PD-1 blocking antibodies target nonredundant pathways, they are currently employed as monotherapy or in combination leading to encouraging and significant clinical benefits in melanoma patients [5,6]. On the other hand, ACT consists of the transfer of a large number of autologous antitumor T cells in lymphodepleted patients [7], where Tumor Infiltrated Lymphocytes (TILs) are collected and expanded ex vivo before their reinfusion in the patient [8,9]. However, TILs activity is often compromised due to the tolerogenic tumor microenvironment. Therefore, a new ACT protocol involving the isolation of autologous circulating T cells which are modified ex vivo to acquire antitumoral activity have been developed [10]. In the last two decades, these therapies have demonstrated durable clinical responses in patients affected by several cancer types [11,12,13,14,15,16,17]. Nevertheless, only 30% of the patients respond to these promising therapies, and to date, only a few tumor types with high mutational load (melanoma, smoking-induced lung cancer and bladder cancer) are suitable for immunotherapy [18]. The immune tolerance and T cell exclusion orchestrated by the tumor microenvironment is thought to be the leading cause of immunotherapy failure with innate immunity playing a central role.

Differently from adaptive immunity that recognizes specific nonself-antigen through a large repertoire of rearranged receptors, innate immunity senses the tumor microenvironment and responds to a variety of less-specific danger signals (pathogen-associated molecular patterns (PAMPs) and damage-associated molecular patterns (DAMPS)) released from the affected cells [19]. The innate immune system can directly inhibit tumor progression [20] by engaging tumoricidal activity. For example, natural killer cells (NKs) are able to lyse tumor cells through the recognition of tumor-derived antigens or cell surface stress molecules [21]. Moreover, polymorphonuclear granulocyte-like neutrophils and eosinophils mediate antitumor activity through the antibody-dependent cellular cytotoxicity (ADCC) or antibody-dependent cellular phagocytosis (ADCP) [22]. Similarly, macrophages can directly participate in tumoricidal activity through ADCC and ADCP [23].

The innate immune cells participate also indirectly in antitumoral processes by recruiting adaptive immune cells, mainly T cells, through the release of various inflammatory cytokines. T cell activation is not an autonomous process and requires antigen presentation from specialized cells within a proinflammatory environment. In this context, DCs and Macrophages are pivotal players contributing to the tumor microenvironment inflammatory landscape. Importantly, T cell infiltration at the tumor site has been proposed as a positive prognostic marker in immunotherapy [24]. T cell recruitment in the tumor microenvironment is orchestrated by a large set of chemokines and cytokines [25] especially produced by myeloid lineages component of the innate immunity such as DCs [26] and macrophages [23]. Therefore, tackling the innate immunity has become an attractive therapeutic opportunity to improve the efficacy of cancer immunotherapies [27].

Indeed, stimulation of innate immunity to treat cancer was first applied in the late 19th Century [28,29]. Although without knowing the cellular mechanism involved, Coley achieved a reduction in tumor growth, and, in certain cases, tumor elimination, by intratumoral injection of inactivated bacteria (*Streptococcus pyogenes* and *Serratia Marcenscens*) [30]. Nowadays, we know that the antitumor immune response in such contexts is triggered by the activation of innate immune response via toll-like receptors (TLRs) recognitions of bacterial particles [31]. The role of innate immunity in tumor development and progression has been deeply investigated for many years; however, over the last decade, the cancer immunology field has centered its focus on the T cell antitumor capacity [27]. It is undeniable that the application of T cell immunotherapy reached unprecedented therapeutic successes in cancer treatment; however, its application is still limited to a few tumor types. In this context, innate immunity is now drawing attention as a potential combinatory target for immunotherapy. Here, we review the contribution of the most abundant myeloid components of the innate immune system on the tumor immune landscape, their impact on the current T cell cancer immunotherapies and the potential opportunities for the development of novel therapeutic strategies.

## 2. Dendritic Cells

**Figure 1:**
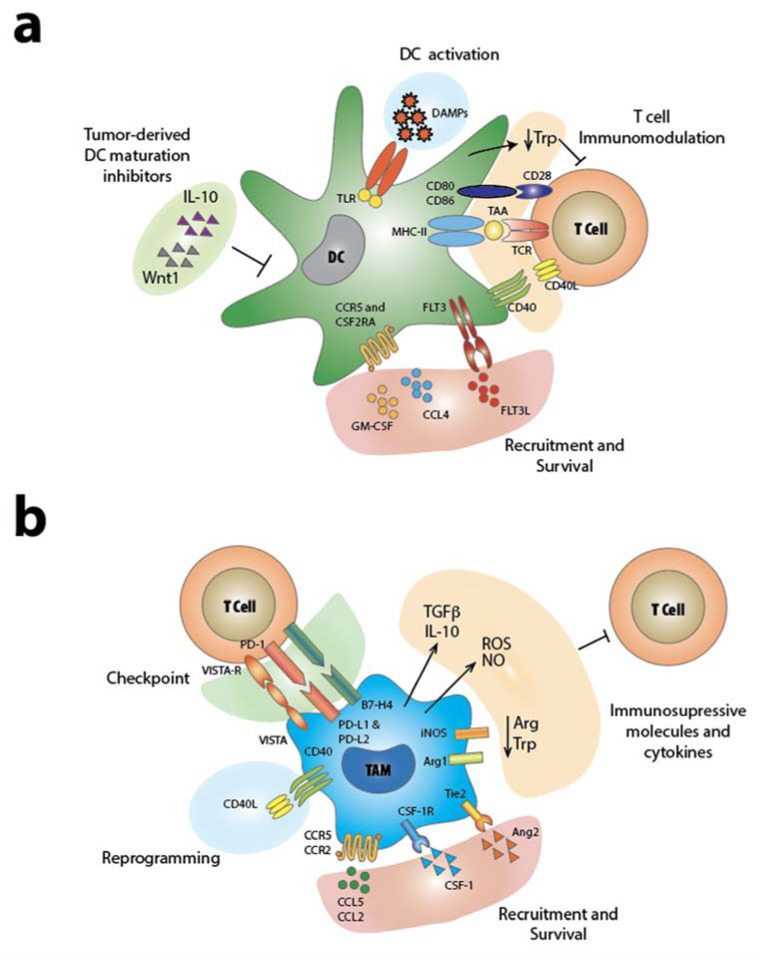
Cross-talk between tumor microenvironment–innate immunity–T cell. (**a**) Dendritic cells (DCs). DCs are recruited in the tumor microenvironment through a series of cues released in the tumor stroma. There, cancer cells produce a series of cytokines that push DCs toward a tolerogenic phenotype. On the other hand, when DCs are activated by DAMPs through their toll-like receptors (TLRs), they mature and they sustain T cell activation and function. (**b**) Tumor-associated macrophages (TAMs). TAMs generally display an M2 immunosuppressive phenotype. They are recruited by various cytokines in the tumor microenvironment where they exploit their immunosuppressive function on T cells through different mechanisms: release of tolerogenic cytokines and checkpoint molecules.

Dendritic Cells (DCs) constitute a heterogeneous group of specialized APC, whose functions are integrated into both the innate and the adaptive immune responses [32]. Their ability to capture, process and present antigens are necessary for the initiation of antigen-specific immunity and, at the same time, for the induction of immune tolerance [33,34]. In the absence of inflammatory stimuli, DCs are defined as immature or tolerogenic. In this state, DCs express low levels of costimulatory and immunoenhancing molecules such as CD40, CD80 and CD86 and contribute to the immune tolerance [35]. Immature DCs are known to infiltrate the tumor microenvironment [33,36] inducing tolerance and anergy of tumor-specific T cells [37,38]. Furthermore, tolerogenic DCs along with anti-inflammatory stimuli like TGF-β can increase immunosuppressive regulatory T cells (Treg) population by conversion of naïve T cells or by the expansion of preformed Treg [39]. Conversely, in the presence of inflammatory stimuli, bacterial or viral-derived products or by ligation with specific receptors (e.g., CD40) DCs undergo maturation becoming a potent stimulator of adaptive immune cells. Activated DCs express costimulatory molecules and chemokine receptors and are able to prime T cells and trigger T cell killing activity against pathogens and cancer cells (Figure 1a). [40] Therefore, DCs have the potential to generate and modulate the antitumor response by recruiting and activating adaptive immunity [41]. Indeed, while dendritic cells are found to be a small cell population in both lymphoid organs and tumor microenvironments, their manipulation hides a great potential for cancer immunotherapy [34].

Notably, conventional anticancer therapies influence immune cell recruitment and function and their efficacy is often dependent on DCs activation. For example, chemotherapy, radiation and cryoablation therapy can promote immunogenic cell death [42] and antitumor immunity by different mechanisms orchestrated by DCs [43,44,45]. Dying cancer cells are characterized by the expression of the “eat-me” signal calreticulin that is required for DC-mediated phagocytosis and consequent induction of antitumor immunogenicity [46]. Furthermore, tumor cell death leads to the release of immunostimulatory molecules such as ATP and Annexin A1 able to recruit DCs in the tumor microenvironment [47,48]. Consequently, DCs accumulation enhances tumor-associated antigen (TAA) cross-presentation and increases the recruitment of TAA-specific CD8^+^ cytotoxic T cells in the tumor [49].

**Figure 2:**
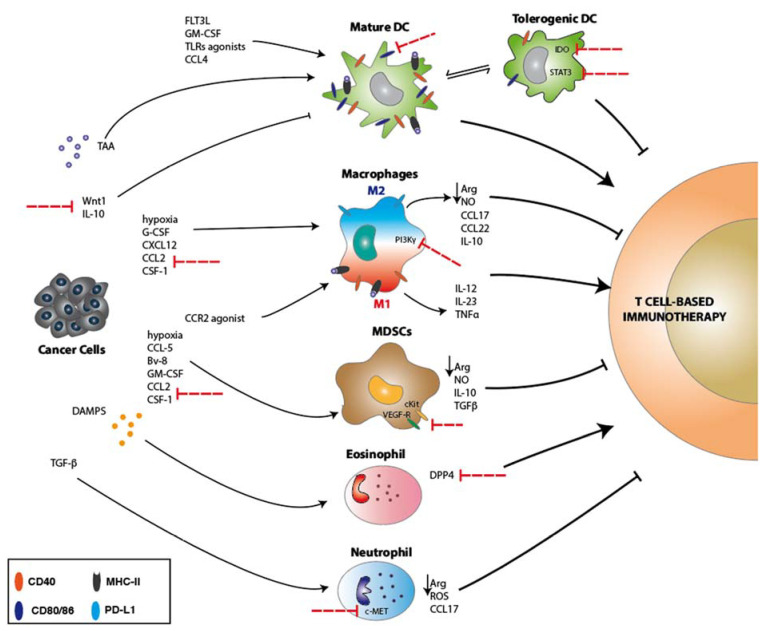
Direct and indirect contribution of innate immune cells in T-cell-based immunotherapy. Innate immune cells can modulate T-cell-based immunotherapy (immune checkpoint blockade (ICB) and adoptive cell therapy (ACT)) through different mechanisms. Depending on the maturation status DCs can boost or inhibit antitumor T-cell-based functions. Several approaches are aimed to promote DC maturation or to inhibit DC tolerogenic activity. Notably, macrophages show, as DCs, a dichotomous behavior in modulating immunotherapy efficacy. Their role is depending on their phenotype, M1 or M2, and several therapies point to skew the differentiation process towards the more antitumoral M1 phenotype. On the other hand, myeloid-derived suppressor cells (MDSCs) participate in mounting immunosuppression in the tumor microenvironment by releasing different cytokines and metabolites that inhibit T cell function. Furthermore, granulocytes, like eosinophils and neutrophils, participate in tuning the T cell activity in combination with classical immunotherapies. Some examples of activator or inhibitory molecules produced by innate immune cells are shown. Red dotted arrows indicate relevant therapeutically targeted molecules aiming to boost T-cell-based immunotherapies.

Besides conventional anticancer therapies, new strategies aimed to exploit DC functions are currently pursued. Tumor vaccines, based on the administration of specific cytokines or/and adjuvants promoting DC activation and thus T cell priming against tumor antigens, have shown promising results in preclinical tumor models. Cytokines such as the FMS-like tyrosine kinase 3 ligand (FLT3L) or the granulocyte–macrophage colony-stimulating factor (GM-CSF) are able to recruit and activate DCs in the tumor microenvironment [50,51] and are currently being tested in different clinical trials as mono or combinatorial therapy (Figure 1a and Figure 2) [52]. Moreover, cancer cells can impair DC maturation by the secretion of soluble molecules, such as Wnt1, or cytokines like IL-10, which activates pro-oncogenic signaling pathways (e.g., STAT3). In this context, agents blocking Wnt1 or STAT3 signaling have shown to relieve immunosuppression at the tumor sites and are currently evaluated in clinical trials for lung adenocarcinoma and hematological malignancies [53,54,55].

Other strategies adopted in cancer therapy aim to block the DC immunosuppressive functions. In the tumor microenvironment, DCs express the enzyme indoleamine 2,3-dioxygenase (IDO) which depletes the amino-acid tryptophan, an essential nutrient required for proper T cell function (Figure 2). Combinatorial targeting of IDO immunosuppressive role with ICB has been clinically tested and has emerged as promising cancer therapy for a variety of cancer types (e.g., melanoma, ovary, breast, myelodysplastic syndrome (MDS)) [56].

Moreover, DC proliferation and infiltration are promoted by immune checkpoint blockade (ICB) therapy, unleashing antitumor adaptive immunity [57]. Interestingly, DCs express PD-L1 making them a direct target of ICB. For example, it has been shown that PD-L1 blockade in DCs improves T cells priming and overall survival of lung and renal carcinoma patients [58].

Conversely, tumors poorly infiltrated by DCs result resistant to ICB therapy [59]. The transfer of in vitro preactivated DCs improves ICB efficacy in nonresponsive preclinical models of melanoma [60]. This evidence has led to the development of novel strategies aimed to improve ICB through the chemokine-driven recruitment of endogenous DCs in the tumor microenvironment. The administration of an engineered fusion form of the chemokine CCL4 targeting the tumor stroma enhances both DCs and T cell immune infiltration and markedly boosts the responsiveness to ICB in poorly T-cell-infiltrated tumors (cold tumor) [61]. Indeed, CCL4 is an important chemokine involved in the recruitment of DCs in the tumor microenvironment through its receptor CCR5 and the lack of CCL4 has been described as a hallmark of cold melanoma tumor [62]. In lung carcinoma patients the expression of a mutated variant of EGFR in the tumor stroma leads to a drastic reduction of intratumoral DCs and consequent reduction of T cells infiltration. Treatment with GM-CSF in combination with the EGFR receptor inhibitor gefitinib rescued the DC population and ameliorated the response to ICB therapy [63,64,65]. Moreover, activation of DCs via administration of toll-like receptors (TLRs) agonists has led to encouraging results when combined with ICB. TLRs are expressed in DCs and other innate immune cells such as macrophages. TLRs recognize pathogen-associated molecular patterns derived from microbes, damaged or necrotic cells, and trigger both innate host defense responses (phagocytosis, inflammation) and antigen-specific adaptive immunity [66] (Kawasaki and Kawai, 2014). Congruently, melanoma patients showed a better clinical response and DCs infiltration when ICB was combined with TLR9 agonist treatment [67].

However, the application of DC-based strategies in cancer immunotherapy is not only restricted in the context of ICB but has been successfully applied in T-cell-based cancer therapy [68]. Tumor-resident DCs are required for proper trafficking of adoptively transferred T cells (ACT) and their scarcity in the tumor stroma limits the therapeutic efficacy of transferred tumor-specific T cells in different preclinical models [69]. Accordingly, the paucity of DCs in the stroma of pancreatic tumors has been associated with their unresponsiveness to immunotherapy. DC mobilization and activation via FLT3 administration elicit CD8 T cell priming and an effective antitumor response in an inducible experimental model of pancreatic cancer [70]. CD8 T cell priming and activation require the interaction with mature and activated DCs to mediate antitumoral and antipathogens activities. facilitating CD8 T cell–DC interactions via overexpression of the C-C chemokine receptor type 4 (CCR4) in CD8 T cells, enhance T cell antitumor responses against pancreatic tumor models [71,72].

As described above, DCs actively participate in the priming and activation of adaptive immunity through direct cell–cell interaction. However, the DC’s role is not limited or restricted at the first steps of T cell activation process, but continues with significant contribution during T cell response with the release of cytokines (e.g., tumor necrosis factors, TNFs) and metabolites sustaining T cell function (Figure 1a and Figure 2) [73].

Importantly, tumor immunotherapy aims not only to kill cancer cells, but also to mount a long-term protective immunity mediated by memory CD8 T cells. In this context, DCs also play a central role in the reactivation of tumor-specific memory T cells, ensuring not only short-term antitumor response but also long-term antitumor immunization [74,75]. All this evidence places DCs as a central hub of the antitumor immune response, being capable to orchestrate the complex multifaceted function of adaptive immunity and making them an attractive target for future combinatorial therapies aimed to enhance the efficacy of current T-cell-based immunotherapy.

## 3. Macrophages

Macrophages are multifunctional components of the innate immunity and consist of different subgroups with different maturation stages and tissue localization [40]. They are the primary defense against pathogens where they exploit their function through phagocytosis and the production of inflammatory cytokines [76]. Indeed, in infection context macrophages are polarized toward M1-phenotype through toll-like receptors (TLRs) and Th1 cytokines (e.g., interferon-γ; IFN-γ) stimulation. M1 macrophages produce proinflammatory cytokines such as IL-12, IL-23 and TNFα and increase their antigen-presenting capacity by boosting the expression of major histocompatibility complex class II (MHC-II) and costimulatory molecules [77]. Conversely, Th2 cytokines, such as IL-4 and IL-13, polarize the macrophages toward M2-phenotype. M2 macrophages have a pronounced anti-inflammatory function through the production of IL-10 and immunosuppressive metabolites of adaptive immunity and they are strongly involved in tissue remodeling and angiogenesis [77,78]. These two subgroups of macrophages participate during two distinct phases of a pathogen infection: the acute phase, where M1 macrophages play a key role in mounting the immune response, and the resolution phase, where M2 macrophages participate in dumping the immune response and tissue repair.

Tumor-associated macrophages (TAMs) are an important component of the immune landscape of tumor microenvironment and normally present M2 polarization. They are recruited into the tumor microenvironment by different chemokines such as CSF-1, G-CSF, CXCL12/SDF-1 and CCL2/MCP1 secreted by stromal and tumor cells [79]. These recruitment factors are specially produced within the hypoxic regions of the tumor (core) that in turns lead to the preferential localization of macrophages in the hypoxic microenvironment [80]. In hypoxic conditions, the activation of hypoxia-inducible factors (HIFs) in macrophages resulted in the selective promotion of the M2 immunosuppressive phenotype with consequent inhibition of T cell-mediated adaptive immunity [81]. Moreover, TAMs help tumor establishment by secreting factors that promote proliferation, angiogenesis, dissemination and distant site colonization [76] and their abundance in the tumor stroma correlates with a poor prognosis of the disease progression [82].

TAM can directly hamper T cell adaptive immunity. Indeed, high expression of arginase-1 (Arg-1) in M2 macrophages leads to the depletion of L-arginine in the tumor microenvironment that is required for T cell fitness and antitumor activity [83,84]. Congruently, mice engrafted with a murine cell line of lung carcinoma have enhanced response to immunotherapy when treated with an arginase inhibitor [85]. Nitric oxide synthase 2 (NOS2) is highly expressed in M2 macrophages and participates in the degradation of L-arginine and the generation of nitric oxide (NO) [86]. Moreover, NO has a direct effect on T cell activation and the use of NO-scavenger has been shown to enhance T cell response to tumor vaccination (Figure 1b) [87]. However, recent studies suggest that the effect of NOS2 in the tumor microenvironment is context-dependent and, in certain cases, can enhance T cell antitumor response [73,88] via induction of vascular cell adhesion molecule 1 (VCAM1) in the tumor endothelium and consequently increased accumulation of adoptively transferred CD8^+^ T cells [88]. Importantly, CD14^+^ or CD68^+^ macrophages express immune checkpoint ligands that can directly interact with T cells and hamper their antitumor activity [84]. Congruently, TAMs isolated from hepatocellular carcinoma (HCC) patients showed enhanced expression of PD-L1, which correlated with patient disease progression and mortality [89]. Moreover, recent studies have demonstrated that the efficiency of anti-CTLA-4 checkpoint therapy in prostate cancer patients was hampered by the presence of CD68^+^ macrophages expressing PD-L1 or V-domain Ig suppressor of T cell activation (VISTA). Together with changes in the macrophage population, patient nonresponders to anti-CTLA-4 therapy showed a higher proportion of CD4^+^, CD8^+^ T cells [90]. Notably, CD68^+^ macrophages expressing PD-L1 or VISTA displayed an M2 polarization characterized by increased expression of CD163 and Arg1. Another immune checkpoint ligand, B7-H4, is highly expressed in TAMs and strongly inhibits IL-2 production and T cell proliferation [91]. Indeed, TAMs present in various human tumor types display high expression of PD-L1 and/or B7-H4 (Figure 1b). However, the contribution of these TAM checkpoint ligands to the overall immunosuppression occurring in the tumor microenvironment still needs to be fully elucidated [23]. In this context, ICB is often not sufficient to overcome the macrophage-induced T cell immunosuppression [92]. An increased body of evidence indicates that TAMs can directly suppress T cell tumor infiltration [84] and thus strongly hinder the efficacy of cancer immunotherapy [93,94]. ICB effectiveness has been successfully achieved when combined with inhibitors of macrophages recruitment in the tumor microenvironment. Therapies combining CCR2 or CSFR-1 antagonists with anticheckpoint antibodies have shown promising results in various cancer models refractory to standard ICB (Figure 2) [84,92]. In these systems, TAM depletion leads to an increased T cell tumor infiltration and thus to an enhanced response to immunotherapy [95] for which several cellular and molecular mechanisms have been proposed [84]. One interesting mechanism that could be involved in the T cell immune exclusion arises from recent studies showing that TGFβ, abundantly produced by TAM, restrains T cell infiltration in both colorectal cancer mouse models and metastatic urothelial cancer patients [96,97].

Finally, TAMs can suppress adaptive immunity through the recruitment of immunomodulatory cell populations in the tumor microenvironment such as myeloid-derived suppressor cells (MDSCs) and regulatory T cells [98,99,100].

The importance of macrophages in modulating antitumor immune response has led to the development of several strategies aimed to enhance the efficacy of T cell immunotherapy. In both preclinical and clinical settings, better efficacy has been observed when classical immunotherapies were combined with macrophage-targeting strategies. This evidence indicates that the major contribution of macrophages in tumor immunosuppression is exerted through the inhibition of adaptive immunity. Some of the approaches nowadays tested in clinical trials are aimed to reprogram macrophages toward M1 polarization and thus toward a more proinflammatory phenotype. Targeting the TNF superfamily member CD40, expressed both by macrophages and DCs, with agonist antibodies have been successfully used to increase antigen-presenting capacities of APCs and elicit tumor-specific T cell priming and activation [101]. Even though the therapeutic contribution, in certain cases, seems to be attributable to DCs [73] and in other to macrophages [102], this approach is currently tested in different clinical trials in combination with checkpoint blockade immunotherapy in metastatic lung and melanoma patients. An alternative strategy to reprogram macrophages involves the targeting of their metabolism through the inhibition of PI3Kγ signaling, which has been shown to be an important molecular hub orchestrating macrophages immunosuppressive activity (Figure 2) [103]. Indeed, inhibition of PI3Kγ results in macrophage reprogramming and increased T cell response [84]. This therapeutic approach is currently tested in clinical trials in combination with checkpoint blockade immunotherapy in refractory Hodgkin lymphoma patients.

However, the most explored strategies at the moment are targeting macrophage recruitment and survival. As mentioned previously, antibodies against CCL2/CCR2 and CCL5 have been employed in preclinical models in combination with checkpoint blockade immunotherapy with encouraging results in several tumor types [100,104,105]. Indeed, CCR2 antagonists are currently tested in patients with advanced melanoma in combination with anti-PD1 or PDL1 therapy. Other pathways regulating macrophage recruitment involved the CXCL12/CXCR4 and angiopoietin2 (ANG2)/TIE2 axes. These therapies especially affect TIE2^+^ TAMs that have been shown to be involved in tumor angiogenesis and malignant progression [106]. ANG2 targeting is currently tested in combination with anti-PD1 therapy in advanced melanoma, ovarian, renal or colorectal carcinomas [84]. TAM abundance is also modulated with novel therapies acting on cell survival. Antagonists acting on CSF/CSFR1 strongly induce apoptosis in TAMs and influence T cell infiltration and response to both ICB and ACT (Figure 2) [84]. Several clinical trials are currently conducted in patients with advanced solid tumors and promising results have been achieved in the treatment of nonresponsive pancreatic cancer patients [84].

Globally, these data indicate that modulation of macrophage recruitment, survival and function can represent a valuable target to improve T cell immunotherapy and further combinatorial approaches possibly will lead to an overall improvement of immunotherapy.

## 4. Myeloid-Derived Suppressor Cells (MDSC)

Myeloid-derived suppressor cells (MDSC) are defined for their ability to suppress T-cell-mediated immune responses, to support tumor progression and to promote a more invasive and malignant phenotype [107]. They represent a heterogeneous immune cell population originally defined by the expression of CD11b and Gr1 markers. However, additional markers associated with their immunosuppressive functions have been identified, such as CD80, CD115 (CSF-1 or M-CSF-receptor), CD124 (IL-4 receptor alpha chain) [107].

MDSCs are composed of two morphologically defined subpopulations: monocytic MDSC (M-MDSC), expressing CD11b^+^ Gr1^+^ Ly6G^-^ Ly6C^high^ markers, and polymorphonuclear (PMN-MDSC), expressing granulocytic/neutrophil-like markers such as CD11b^+^ Gr1^+^ Ly6G^+^ Ly6C^low^ [108]. MDSCs are mobilized from the bone marrow and recruited into the primary tumor site through various chemo-attractant factors such as M-CSF, GM-CSF, CCL-2 (MCP-1), CCL-5 (RANTES), Bv8 [109,110], which are particularly secreted in hypoxic regions of the tumor (Figure 2) [111].

Functionally, they have been characterized for their capacity to suppress T cell-mediated immune responses. Different immunosuppressive mechanisms have been described, including the release of tolerogenic cytokines and nitric oxide (NO), the generation of reactive oxygen species (ROS), and induction of L-Arginase. As a result of the activity of these enzymes (iNOS and Arg2), the tumor microenvironment is depleted of L-arginine, impeding T cell proliferation, activation and antigen recognition, and finally favoring a tolerogenic tumor milieu (Figure 2) [112].

MDSCs can directly hamper adaptive immunity through the release of type 2 cytokines, such as IL-10 and TGF-β, or indirectly by promoting M2 polarization of macrophages, hindering the release of type 1 cytokines, such as IL-12.

More recently, MDSCs have been studied for their influence on anticancer immunotherapies. The presence of MDSCs in both tumor infiltrate and blood circulation correlates to reduced overall survival (OS) and to worse outcomes in breast, lung and colorectal cancer patients. These immunosuppressive cells are preferentially recruited in advanced tumor stage and size, leading to a negative contribution in melanoma patient response to immunotherapy [113,114]. To improve immunotherapy efficacy and counteract MDSC tumor-promoting and immunosuppressive functions, in the last years, novel therapeutic strategies have been developed acting at different levels of MDSC contribution. For example, it has been shown that all-trans retinoic acid (AT-RA) is able to reduce the MDSC number in circulation (expansion) [115]. Moreover, the blockade of VEGF-receptor, c-Kit signaling, or upon cytotoxic agent treatments, as gemcitabine and 5-fluorouracil (5FU), specifically induce apoptosis in MDSCs (Figure 2) [116,117].

Other strategies involve the inhibition of their recruitment in the tumor microenvironment, such as interventions targeting the CCL2 axis by CCR5 receptor antagonists. Indeed, in a mouse model of neuroblastoma the blockade of CSF-1 (M-CSF)/CSF-1R signaling inhibited MDSC recruitment and improved the response to anti-PD-1 therapy [118]. On the other hand, targeting the MDSC interference on T-cell-mediated immune response via the inhibition of phosphodiesterase showed to act synergistically with PD-1 blockade in preclinical models as in ongoing clinical trials, similarly to the HDAC inhibitor entinostat [119,120],.

Another interesting aspect that has been recently explored is the use of MDSCs as predictive markers for response to ICB in metastatic melanoma patients, where patient responders to ipilimumab showed a significantly lower fraction of circulating MDSCs as compared to the nonresponders [121].

Furthermore, preclinical studies have shown that the combination of MDSC depletion strategies with ICB boosts the infiltration of adaptive immune cells, reduces metastatic progression and increases survival rate in preclinical models of breast, lung and renal cell carcinoma [119]. Similarly, the elimination of PMN-MDSC CXCR2^+^ trafficking with the small-molecule inhibitor SX-682 of CXCR1/CXCR2 receptors resulted in an improved response to ACT in a syngeneic model of squamous cell carcinoma (SCC) [122].

Globally, targeting MDSCs’ contribution to tumor progression could help to reshape the tolerogenic tumor microenvironment and could improve T-cell-based cancer immunotherapies. However, MDSCs share several targets with other myeloid components of the tumor microenvironment and the response to a specific therapy still needs to be clearly attributed to the heterotypic cellular component of the tumor stroma.

## 5. Neutrophils and Eosinophils

Neutrophils are the most abundant population of white blood cells in humans, playing important roles in the inflammatory process and primary immune response [123]. They have been mainly described as short-living immune cells and for their role in the clearance of extracellular pathogens. However, this concept has been overstepped with the discoveries of their capacity to live much longer than expected and to produce several key cytokines, chemokines and Fc receptors [124]. Nowadays, evidence has demonstrated important contributions of neutrophils both in the promotion and inhibition of tumor development and growth [125]. This dichotomy suggests that neutrophils are composed of diverse cellular subsets. However, the identification of these subgroups has been trivial due to the lack of robust surface cell markers [125], limiting their characterization of physical properties and functionality [126]. Neutrophils are thought to support tumor growth and progression. Higher neutrophils to lymphocytes ratio represent a poor prognostic marker for a variety of cancer types such as lung and breast cancer [126]. Indeed, neutrophils can support metastatic colonization in a mouse model of breast cancer, orchestrating the formation of the premetastatic niche in the lung [127]. Neutrophils are recruited in the tumor microenvironment by a series of attracting CXC cytokines released by cancer cells [124]. Furthermore, neutrophils are able to sustain tumor progression through the release of proangiogenic factors such as VEGF and MMP9 [128,129] and, by maintaining an immunosuppressive tumor microenvironment through the expression of the arginine-consuming enzyme Arg1 and ROS production [130]. Arginine depletion and ROS directly inhibit T cell antitumor activity and, therefore, neutrophils can negatively impact the efficacy of T-cell-based cancer immunotherapy (Figure 2).

As mentioned above, neutrophils can also exert cytotoxic activity against tumor cells. During the first phases of tumor formation when the immunosuppressive environment is not yet completely established [125] neutrophils recognize and kill cancer cells through ADCC. Neutrophils contribute to tumor control and tumor eradication also by regulating the activation and function of adaptive immune cells like CD4 or CD8 T cells [124,131]. Indeed, neutrophils also function as APC and thus can directly interact with T cells, supporting a robust antitumor T cell activity. [132,133,134,135,136]. Conversely, neutrophils can recruit and activate Treg cells following the secretion of cytokines like CCL17 and promoting an immunosuppressive environment (Figure 2) [137]. However, in the tumor microenvironment neutrophils, as well macrophages, exert generally a tolerogenic activity and consequently hampering the effectiveness of T-cell-based immunotherapy. In this context, TGFβ can polarize neutrophils toward an immunosuppressive phenotype and hinder CD8 T cell tumor infiltration [133]. Blocking TGFβ in the tumor boosts CD8 T cell infiltration and CD8 T cell-dependent antitumoral response [133]. On the contrary, IFNβ has been shown to promote neutrophil antitumor activity and recruitment in the tumor microenvironment [125,138].

Blocking the immunosuppressive function of tumor-infiltrating neutrophils has shown to improve the effectiveness of T-cell-based immunotherapies, such as ICB and ACT. In a murine model of melanoma, the inhibition of the receptor tyrosine kinase c-MET (HGF-R, hepatocyte growth factor receptor) reduces neutrophil recruitment in the tumor microenvironment and in the draining lymph nodes in response to immunotherapy (Figure 2). Consequently, c-MET inhibition enhances the response to ICB and ACT [139]. Meanwhile, the inhibition of the fatty acid transporter protein 2 (FATP2) abrogate neutrophil immunosuppressive activity and response to ICB [140]. In addition, under certain stress conditions, neutrophils can release DNA-based structures, named neutrophil extracellular traps (NETs), which enable neutrophils to capture and kill extracellular pathogens [141]. This cell death phenomenon is nowadays called NETosis [142] and it represents a mechanism adopted by neutrophils to modulate adoptive T cell immunity. NETs are able to interact with DCs enhancing their maturation [143,144], resulting in the upregulation of costimulatory molecules such as CD40, CD80/CD86 and MHCII and proliferation of CD4 T cells [145]. Furthermore, NETs can directly prime T cells leading to increased expression of activation markers such as CD69 and CD25 [146]. Notably, T cells primed by NETs were able to respond to specific antigens even in the absence of costimulatory signals. It is worth noting that this scenario is common in the tumor microenvironment, and, therefore, NETosis can be exploited to induce or to modulate the activation of tumor-specific T cells. However, neutrophilia and NETs formation support disease progression and metastasis formation in lung and colon cancer, and blocking NETosis resulted in decreased cancer cell dissemination [147]).

Furthermore, it has been shown that NETs can protect cancer cells against T cells and NK cell-mediated cytotoxicity [148]. Tumors cells are able to recruit neutrophils and to sustain NETosis through the secretion of neutrophils chemoattractants, like ligands of the chemokine receptors CXCR1 and CXCR2 [149]. NETs coated tumor cells were physically protected against adaptive immune cells, impairing T cells and NK cells motility and blocking them from reaching the tumor. Notably, pharmacological inhibition of NETosis sensitizes tumors to ICB therapy [149].

This evidence highlights the potential therapeutic opportunities in targeting neutrophils combined with immunotherapies. However, neutrophil contribution to tumor development and progression still needs to be fully elucidated [124] and neutrophil-targeting therapies capable of boosting T-cell-based immunotherapy have not yet reached clinical applications.

Eosinophils participate in tissue repair processes and in the immune response against parasites. Moreover, eosinophils play a role in several diseases like allergic asthma, autoimmune disorders and cancer [150,151]. Although eosinophils were described in tumor samples at the end of the 19th century and are known to infiltrate several tumor types [152], their function in tumor progression and in the tumor microenvironment is still subject of investigation [153]. Studies have shown increased eosinophil count upon ICB therapy in melanoma, prostate and lung cancer patients which positively correlated with the disease prognosis [154,155,156,157]. The release of DAMPS by necrotic cells in the tumor stroma is proposed to be responsible for eosinophil recruitment (eosinophilia) [158]. Indeed, eosinophil infiltration in the tumor microenvironment of melanoma patients treated with ICB positively correlates with increased TILs infiltration and better patient outcome [159]. Activated eosinophils are able to remodel and normalize tumor vasculature, promoting T cell infiltration and boosting ACT efficacy [160]. In addition, eosinophils can support antitumor immunity modulating macrophage polarization. Notably, proinflammatory cytokines such as INFγ and TNFα are produced by activated eosinophils. Therefore, when transferred together with T cells, activated eosinophils promote the polarization of TAM toward the M1-like phenotype, contributing to the establishment of a proinflammatory tumor microenvironment [160]. Due to their tumor-homing properties, eosinophils are becoming novel potential targets to improve T-cell-based immunotherapies. In the preclinical mouse model of hepatic, prostate and breast cancer, inhibition of the dipeptidyl-peptidase 4 (DPP4) with the FDA approved drug sitagliptin enhances host eosinophil-mediated antitumoral activity and contribute to ICB efficacy [161].

These promising studies can pave the way for new therapeutic strategies aimed to enhance T-cell-based immunotherapy through DPP4 inhibition (Figure 2) [162].

## 6. Contribution of Aging on Innate Immunity and T-Cell-Based Immunotherapy

An important aspect that has been poorly explored but that bridges immunotherapy response to the balance between lymphoid and myeloid compartments, is age. To some extent, cancer can be considered as an age-related disease [163]. Notably, cancer primarily occurs in the elderly and for most types of this disease, incidence rates progressively increase with age (0–24 years old, 1%; 25–49 years old, 10%; 50–74 years old, 53%; 75+ years old, 36%; Cancer Research UK) [164]. In this context, aging is taking part to the erosion of immune competence, a process called immunosenescence, that manifests robustly in the component of the adaptive immunity [165], as result of thymic atrophy [166], a decrease of naïve T cell [167], reduction of memory function [168] and decrease of TCR repertoire [169]. Moreover, aging strongly affects hematopoietic stem cell (HSC) potential [170] and skews them toward myeloid lineages [171]. This process called myeloid bias dramatically reduces the efficiency of T cell production and thus compromises immune reconstitution and immunosurveillance [172]. Similarly to aging, chemotherapy produces substantial damages to the immune system by affecting HSC function, leading to myeloid bias differentiation and subsequent immunosuppression [173]. Therefore, aged patients or patients that have undergone several rounds of chemotherapy may have a reduced response to immunotherapy. Indeed, in lung cancer, prior chemotherapy exposure was associated with reduced benefit from PD-L1 blockade therapy, which was higher in chemotherapy-naïve patients [174].

Moreover, aging leads to a gradual decline of the capacity of our adaptive immune system to recognize and eliminate the fret and it predisposes patients to a higher risk of infection and tumor incidence. These observations raise the need to change the focus of immunotherapies from a T cell centric to a broader view including the innate immune compartments. Notably, the expansion of the innate immune compartment during aging or chemotherapeutic treatment plays an important role in the success of T-cell-based immunotherapy. One important example is the expansion in aged individuals of the heterogeneous population of immature MDSCs [175]. This myeloid population conferees tumor tolerance and is generally expanded in cancer and inflammatory states [176]. Congruently, old mice have a higher proportion of M2 macrophages both in the spleen and in the bone marrow [177], while neutrophils in aged patients produce more anti-inflammatory cytokines [178]. Taken together, all these phenomena contribute to the general inhibition of adaptive immunity response. In this context, another possible strategy to tackle innate immunity-induced immunosuppression in aged or chemotherapy-treated patients is to manipulate HSC fate toward lymphoid lineage [179], reducing the myeloid compartments and, at the same time, preserving the lymphoid compartments. Some approaches targeting HSCs metabolism, like NAD boosting strategies, have been shown to be able to reprogram HSC fate toward lymphoid lineages [180,181] but their application in immuno-oncology still needs to be further investigated. Recently it has been reported that prolonged fasting protects against chemotherapy/aging-induced changes in HSC function in mice, by reducing circulating IGF-1 levels and as a consequence, inhibiting PKA activity [179]. Prolonged fasting, as well NAD boosters supplementation, represents a potentially powerful means to protect against ravages of chemotherapy and aging [179,182,183,184], perhaps allowing higher doses of the drugs or a more efficient application of T-cell-based immunotherapy by preserving the correct balance between lymphoid and myeloid compartments [182].

## 7. Conclusions

The clinical successes of immuno-oncology arise primarily from a T cell-centered vision, which relies on the capacity of infiltrated T cells in controlling and killing tumor cells. However, T cell activation is not a cell-autonomous process and requires the contribution of several cell types of the innate immunity orchestrating complex interactions within the tumor microenvironment. Innate immunity plays a central role in cancer development and progression, both through the remodeling of the tumor microenvironment and establishment of an immunosuppressive environment. The innate immune cell capacity to modulate the adaptive immune response has inspired attempts and trials aimed at hijacking these processes and unleashing a T cell antitumoral response. In the last years, the importance of targeting innate immune cells is recognized by the increasing number of clinical trials for both cancer or autoimmune disorders aimed to harness innate immunity [40]. An interesting approach that has been recently developed contemplates the application of CAR technology to “non-T cell” immune cells such as NK cells and macrophages [185,186]. Interestingly, macrophages have better capacity to penetrate the tumor than T cells. Moreover, the engineered CAR macrophages have direct phagocytic capability against the tumor and display M1 polarization and thus favoring the recruitment and activation of adaptive immunity [186]. All these evidences highlight the necessity to develop holistic approaches capable of dissecting the contribution and interplay of the different immune components of the tumor microenvironment. T-cell-based approaches have been extremely successful for the treatment of a variety of cancer types, but still not applicable to the majority of them.

Future directions of combinatorial therapies include the use of metabolic modulators that can act both on the innate and adaptive immunity compartments [187,188] and, as in the case of NAD boosters, protect against the ravages of aging [189]. In fact, NAD metabolism has been shown to play an important role in macrophage effector function [187] and in supporting their proinflammatory phenotype [190] and, at the same time, modulating antitumor T cell response [188] and protect against T cell immunosenescence [189]. However, these studies still need further investigation in order to better understand the global effect of metabolic modulators on the different components of the tumor microenvironment and exclude possible support to growth or survival of cancer cells, as reported for NAD metabolism in certain hematological malignancies [191,192,193,194].

Thus, further understanding of the contribution of the innate immune compartments to anticancer adaptive immune response will be a key aspect to explore in order to develop combinatorial therapeutic strategies that would expand the clinical response to cancer immunotherapy.
